# Maternal depression and non-specific health complaints in the offspring: a cross-sectional study in Danish primary care

**DOI:** 10.3399/bjgp20X714173

**Published:** 2021-01-26

**Authors:** Bente Kjær Lyngsøe, Dorte Rytter, Trine Munk-Olsen, Claus Høstrup Vestergaard, Kaj Sparle Christensen, Bodil Hammer Bech

**Affiliations:** Research Unit for General Practice, and Department of Public Health, Aarhus University.; Department of Public Health, Aarhus University.; National Centre for Register-based Research, Department of Economics and Business Economics, Aarhus University, Aarhus, Denmark.; Research Unit for General Practice, Aarhus University.; Research Unit for General Practice, and Department of Public Health, Aarhus University.; Department of Public Health, Aarhus University.

**Keywords:** child health, depression, health impact assessment, health status, maternal exposure

## Abstract

**Background:**

Maternal depression has been linked to adverse outcomes in the offspring. Existing literature is mainly based on parental reports, which can be an unreliable source when the parent has depression.

**Aim:**

To explore if maternal depression was associated with daily health complaints and low self-assessed health (SAH) in the offspring.

**Design and setting:**

Participants were 45 727 children from the Danish National Birth Cohort recruited between 1996 and 2002. At 11-year follow-up, mothers and their children were invited to complete a questionnaire. Maternal depression was categorised into: no depression, first-time treatment, continued treatment, post-treatment, and relapse.

**Method:**

Binomial regression was used to estimate the adjusted prevalence proportion ratio (aPPR) of frequent health complaints and low SAH in children of mothers with depression compared to children of mothers without depression.

**Results:**

The prevalence of any daily health complaint was 11.4%, daily somatic complaints 4.1%, daily mental complaints 8.9%, both daily mental and somatic complaints 1.5%, and low SAH 5.3%. Children of mothers with depression (any category) were more likely to report a daily health complaint: first-time treatment aPPR 1.35 (95% confidence interval [CI] = 0.96 to 1.85), continued treatment aPPR 1.59 (95% CI = 1.37 to 1.85), post-treatment aPPR 1.30 (95% CI = 1.20 to 1.41), and relapse aPPR 1.56 (95% CI = 1.35 to 1.79). Children of mothers with depression were also more likely to report low SAH: first-time treatment aPPR 1.58 (95% CI = 0.99 to 2.54), continued treatment aPPR 1.86 (95% CI = 1.51 to 2.28), post-treatment aPPR 1.34 (95% CI = 1.19 to 1.50), and relapse aPPR 1.56 (95% CI = 1.26 to 1.93). Girls had a higher prevalence of mental and somatic health complaints and more often reported low SAH compared to boys.

**Conclusion:**

Treatment of maternal depression was associated with higher prevalence of daily health complaints and low SAH in the offspring at age 11 years. The association was strongest for children of mothers with continued depression or relapse.

## INTRODUCTION

Non-specific health complaints such as headache, abdominal pain, and back pain in pre-adolescents are increasing.^1^^–^^4^ In primary health care, non-specific complaints account for more than two-thirds of reported symptoms.^5^ A rate of 40% has been seen in those aged ≤10 years,^6^ and an average of 27% of 11-year-olds reported multiple non-specific health complaints more than once a week.^3^ A Danish school survey found that 20% of girls and 16% of boys reported experiencing at least one daily emotional symptom.^7^

GPs manage children with non-specific complaints, as well as parents suffering from depression. It is important that GPs be aware of any association between the two. It might be possible that addressing family difficulties related to parental depression could relieve non-specific health complaints in the offspring. In addition, when a GP sees a mother with depression, awareness of possible health complaints in the offspring might benefit the entire family.

Daily health complaints may reduce functioning level and result in excessive school absence,^8^ and increase the risk of somatic and mental illness in adulthood.^9^^–^^12^

Non-specific health complaints have been linked to lower self-assessed health (SAH).^13^^,^^14^ SAH is a subjective measure of wellbeing, used widely in research in adults, and is associated with pain, existing diseases, vitality (that is, feeling energetic and full of life), and mortality.^15^^–^^17^ Low SAH is a strong predictor of disability, increased use of healthcare services,^14^ higher mortality,^18^ and medication prescription later in life.^19^ Previous studies have found poor SAH in adolescence to be associated with multi-illness in early adulthood, and mortality.^20^^,^^21^

Parental mental health problems are closely related to somatic and mental health complaints and the level of SAH in the offspring.^22^^–^^26^ Depression is one of the most common mental illnesses. One in 10 children are estimated to be exposed to maternal depression in any given year.^27^ Most existing research is based on parental reports, which can be unreliable when the parent has depression,^28^^–^^30^ whereas SAH in pre-adolescents has been found to be reliable and remains stable over time.^14^^,^^28^^,^^29^ This study adds results based on pre-adolescents’ own reports. Recent research has found higher past and future use of health care in general practice in pre-adolescents with non-specific health complaints and low SAH.^31^

The aim of this study was to investigate if treatment for maternal depression (categorised into first-time, continued, post, and relapse) is associated with daily health complaints and low SAH in the offspring.

## METHOD

### Design, setting, and population

**Figure 1.**
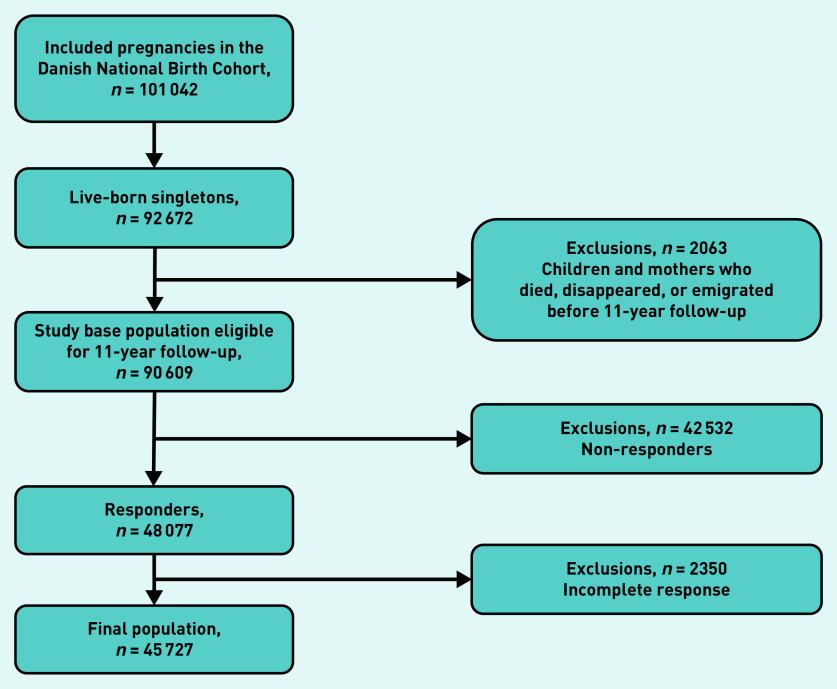
***Flow chart.***

This cohort study included children participating in the Danish National Birth Cohort (DNBC), which has been described in detail elsewhere.^32^ In short, pregnant women were recruited from 1996 to 2002 (*n* =101 042). The mothers were interviewed multiple times during pregnancy and through early childhood. At 11-year follow-up, the mothers and their children were invited to fill in a web-based questionnaire. In the invitation letter, the mothers were informed that it was preferable that children filled out the questionnaire themselves and alone, in order to obtain the most honest answers. Short audiofiles for every question and answer were supplied to enable the participation of children with conditions such as dyslexia. The DNBC included 92 672 live-born singletons. After exclusion of children or mothers who died, disappeared, or emigrated before 11-year follow-up (*n* = 2063), 90 609 children remained eligible for the 11-year follow-up. The final study population consisted of 45 727 children with complete responses to the items described, and their 43 228 mothers (Figure 1).

**Table:** How this fits in

Non-specific health complaints in pre-adolescents are correlated to physical and psychosocial disabilities later in life. Most studies are based on parental reports, which can be unreliable when the parent has depression. This study found that children of mothers treated for depression had a higher prevalence of frequent health complaints, especially children of mothers in continuous treatment. This study adds useful knowledge for the daily management of parents with depression and pre-adolescents with health complaints in primary health care.

### Maternal depression

Maternal depression was identified through records of redeemed antidepressant prescriptions, and through outpatient hospital contacts and inpatient hospital admissions, with depression as the main diagnosis recorded in accordance with the *International Classification of Diseases, 10th Revision* (ICD-10) (Table 1). Thus, the definition of depression was based on treatment of depression, and throughout this research the term depression describes treatment of depression. All information was obtained from the Danish National Prescription Register^33^ and the Danish National Patient Register^34^ for the period 1995–2015. Defining depression from register-based information has been validated elsewhere.^35^ A mother was defined as depressed for 90 days after an outpatient contact and for 180 days after hospital admission. To reduce misclassification, a minimum of two outpatient contacts or prescriptions within a 6-month period were required as a criterion for depression. The authors excluded certain antidepressant medications used for smoking cessation and chronic pain management. Depression duration was calculated from package and dosage of antidepressant medication in accordance with recommendations by the World Health Organization.

**Table 1.** Criteria for exposure and description of exposure

**Exposure measures**	**Specifications**	**Duration of depression**
Antidepressant medications	ATC codes: N06AB03, N06AB04, N06AB05, N06AB06, N06AB08, N06AB10, N06AF01, N06AG02, N06AX03, N06AX06, N06AX11, N06AX16, N06AX21, N06AX22	Duration based on dosage and package of the second prescription
Outpatient contact to a hospital	Main diagnosis of depression according to the ICD-10 classification: F32.0, F32.1, F32.2, F32.3, F32.8, F32.9, F33.0, F33.1, F33.2, F33.3, F33.4, F33.8, F33.9	90 days following a contact
Admission to a hospital	Main diagnosis of depression according to the ICD-10 classification: F32.0, F32.1, F32.2, F32.3, F32.8, F32.9, F33.0, F33.1, F33.2, F33.3, F33.4, F33.8, F33.9	180 days following an admission

*ATC = Anatomical Therapeutic Chemical.* *ICD-10 = International Classification of Diseases, 10th Revision.*

To determine maternal depression status at the time of completion of the questionnaire at 11-year follow-up, the authors included information from registries back to 1995 and categorised maternal depression status into one of the following five exposure groups:

- No depression (reference group): a mother with no events before 11-year follow-up.
- First-time treatment: after the first event, the mother was categorised as first-time treatment for a maximum of 1 year.
- Continued treatment: if the treatment continued for >1 year, the mother was categorised as continued depression.
- Post-treatment: after cessation of treatment, the mother was categorised as post-treatment.
- Relapse treatment: a new event in a mother in the post-treatment group was categorised as relapse for a maximum of 1 year. If the treatment continued >1 year, a mother was categorised as continued depression.

### Health complaints in the offspring

The first outcome of interest was daily somatic and mental health complaints. Somatic health complaints were assessed by four questions in which the child reported the frequency of headache, stomach ache, dizziness, and constipation during the previous 6 months. Mental health complaints were assessed by four questions in which the child reported the frequency of sadness, irritability or bad temper, nervousness, and insomnia during the previous 6 months. For both somatic and mental complaints, the response options were: almost every day; more than once a week; almost every week; more than once a month; almost every month; or, rarely or never. As in previous research on this cohort,^31^ the outcome was reporting the presence of daily and almost every day health complaints. This symptom checklist has previously been found to have high reliability and validity.^36^^,^^37^

### Self-assessed health in the offspring

The second outcome of interest was SAH at 11-year follow-up. In the present study, the question: ‘How do you regard your overall health?’ was used to assess SAH. The response options were: very good, good, fair, and poor. SAH was dichotomised into ‘good’ (very good and good) and ‘low’ (fair and poor). This measure has been found to be a reliable and stable measure over a period of 4 years.^14^

### Covariates

Included covariates were selected a priori based on previous findings, evidence, and availability.^7^^,^^14^ Information on age and sex of the child and maternal age was obtained from the Danish Civil Registration System.^38^ Information on parity (one, two, or greater than or equal to three births) was obtained from the Danish National Patient Register.^34^ Statistics Denmark (https://www.dst.dk/en/Statistik) provided information on civil status of the mother (divorced, married, or unmarried), maternal education (primary/lower secondary, upper secondary, or tertiary) at the year of questionnaire completion. Paternal depression was defined similarly to maternal depression, and the same data sources were used.

Missing values in any of the covariates were included in the analyses as an ‘unknown’ category. Missing information on at least one covariate was seen for approximately 1.3% of the population (both responders and non-responders). In the responder population, the percentage of children with missing values for maternal covariates was approximately 0.8%.

### Statistical methods

All analyses were performed using Stata (version 15), and the significance level was set at 5%. The mean age of the children at the date of questionnaire completion was 11.4 years. Therefore, for non-responders, the authors used the maternal depression status at 11.4 years to describe their exposure status. To address this selection bias, the authors used inverse-probability weighting (IPW) analysis.^39^ A logistic regression model was built to estimate the probability of responding for each child by using the information on all 90 609 cases in the study base population and all variables described in covariates and exposures. The inverse predicted probabilities were used as weights in the IPW analysis. This weighting of the responding population aimed to produce estimates representative of the study base population. Binomial regression was used to estimate the adjusted prevalence proportion ratio (aPPR) of daily health complaints and low SAH levels in children of mothers with depression compared to children of mothers without depression.^40^ Cluster robust variance estimation was applied at maternal level to accommodate any dependence due to inclusion of siblings in the cohort. The association between maternal depression and various offspring outcomes has previously been suggested to differ between boys and girls.^41^ Therefore, the authors investigated possible effect modification by stratifying the analyses by the sex of the child.

## RESULTS

### Participants

Of the 90 609 children in the study base population, 48 077 (53.1%) children responded. Incomplete responses concerning health complaints or SAH were found for 4.9% (Figure 1). Mothers of responders had higher educational level, fewer were depressed, and more were married compared to mothers of non-responders. Fewer of the responding children had fathers with depression, more were girls, and they had fewer older siblings compared to mothers of non-responders (data not shown).

Mothers of responding children with depression had lower educational level, more were divorced, and fewer were married compared to mothers without depression. Children of mothers with depression more often also had a father with depression (Table 2).

**Table 2.** Characteristics of participants in the five exposure groups at the date of questionnaire completion

	**Mothers with no depression**	**Post**	**First-time**	**Continued**	**Relapse**	**Total**
***n* (%)**	38 394 (84.0)	5044 (11.0)	205 (0.4)	969 (2.1)	1115 (2.4)	45 727 (100.0)

**Maternal age, mean SD**	42.2 (4.1)	41.8 (4.6)	41.5 (4.3)	42.0 (4.5)	41.9 (4.6)	42.2 (4.2)

**Child age, mean SD**	11.4 (0.6)	11.4 (0.6)	11.5 (0.6)	11.4 (0.6)	11.5 (0.7)	11.4 (0.6)

**Child sex, *n* (%)**						
Boy	18 353 (47.8)	2390 (47.4)	95 (46.3)	453 (46.7)	504 (45.2)	21 795 (47.7)
Girl	20 041 (52.2)	2654 (52.6)	110 (53.7)	516 (53.3)	611 (54.8)	23 932 (52.3)

**Maternal education, *n* (%)**						
Primary	1818 (4.7)	481 (9.5)	14 (6.8)	93 (9.6)	119 (10.7)	2525 (5.5)
Secondary	13 791 (35.9)	2057 (40.8)	80 (39.0)	394 (40.7)	443 (39.7)	16 765 (36.7)
Tertiary	22 489 (58.6)	2458 (48.7)	111 (54.1)	471 (48.6)	541 (48.5)	26 070 (57.0)
Unknown	296 (0.8)	48 (1.0)	0 (0.0)	11 (1.1)	12 (1.1)	367 (0.8)

**Maternal civil status, *n* (%)**						
Divorced	3577 (9.3)	919 (18.2)	31 (15.1)	179 (18.5)	173 (15.5)	4879 (10.7)
Married	30 932 (80.6)	3442 (68.2)	144 (70.2)	679 (70.1)	797 (71.5)	35 994 (78.7)
Unmarried	3885 (10.1)	683 (13.5)	30 (14.6)	111 (11.5)	145 (13.0)	4854 (10.6)

**Number of older siblings, *n* (%)**						
1	18 410 (48.0)	2379 (47.2)	101 (49.3)	455 (47.0)	562 (50.4)	21 907 (47.9)
2	13 992 (36.4)	1843 (36.5)	81 (39.5)	360 (37.2)	390 (35.0)	16 666 (36.4)
≥3	5992 (15.6)	822 (16.3)	23 (11.2)	154 (15.9)	163 (14.6)	7154 (15.6)

**Paternal depression (yes), *n* (%)^a^**	3704 (9.6)	834 (16.5)	34 (16.6)	169 (17.4)	183 (16.4)	4924 (10.8)

a Due to low numbers, paternal depression was aggregated to yes/no in the table. SD = standard deviation.

### Main findings

**Figure 2.**
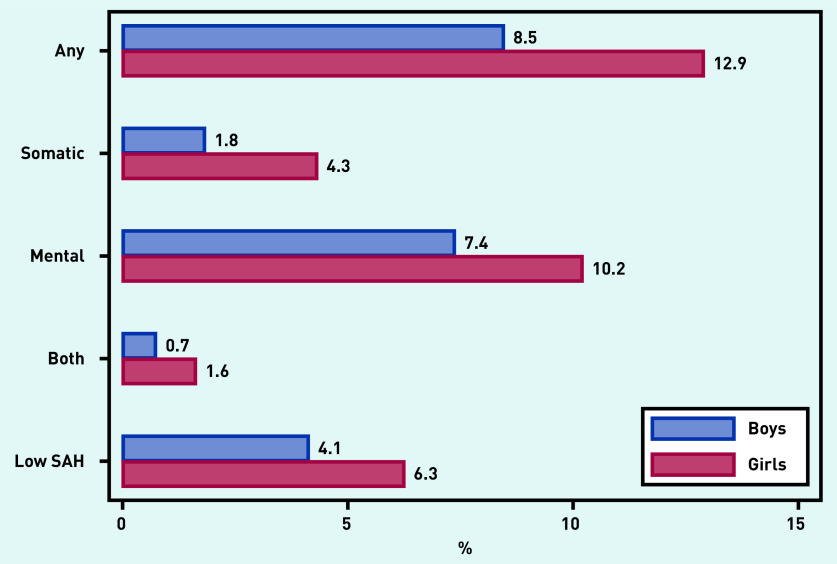
***Prevalence of daily health complaints and low self-assessed health. SAH = self-assessed health.***

The prevalence of any daily health complaint was 11.4%, daily somatic complaints 4.1%, daily mental complaints 8.9%, both mental and somatic complaints 1.5%, and low SAH 5.3% (Table 3). Girls had higher prevalence of health complaints and lower SAH than boys (Figure 2). The most common daily complaints were, in descending order, insomnia, irritability, headache, sadness, stomach ache, nervousness, dizziness, and constipation.

**Table 3.** Adjusted prevalence proportion ratio (aPPR) for reported daily health complaints and poor/fair self-assessed health in children of mothers treated for depression compared to children of mothers not treated for depression^a^

	**First-time treatment aPPR (95% CI)**	**Continued treatment aPPR (95% CI)**	**Post-treatment aPPR (95% CI)**	**Relapse**
**Any complaints** *n* = 5230	1.35 (0.96 to 1.89)	1.59 (1.37 to 1.85)^b^	1.30 (1.20 to 1.41)^b^	1.56 (1.35 to 1.79)^b^
**Somatic complaints** *n* = 1860	1.49 (0.80 to 2.76)	2.01 (1.54 to 2.61)^b^	1.41 (1.22 to 1.63)^b^	1.39 (1.05 to 1.86)^b^
**Mental complaints** *n* = 4062	1.45 (1.00 to 2.09)	1.57 (1.33 to 1.86)^b^	1.27 (1.16 to 1.39)^b^	1.67 (1.43 to 1.94)^b^
**Both complaints** *n* = 692	2.38 (1.06 to 5.35)^b^	2.51 (1.68 to 3.76)^b^	1.39 (1.09 to 1.77)^b^	1.90 (1.26 to 2.87)^b^
**Low SAH** *n* = 2412	1.58 (0.99 to 2.54)	1.86 (1.51 to 2.28)^b^	1.34 (1.19 to 1.50)^b^	1.56 (1.26 to 1.93)^b^

a Reference group is children of mothers without depression.

b Statistically significant data. All estimates are adjusted for child age and maternal age, sex of the child, parity (three groups), maternal civil status (three groups), and education (three groups) at the date of questionnaire completion. Maternal civil status (three groups), education (three groups), and paternal depression (five groups) was defined by the same criteria as maternal depression at the date of the questionnaire. aPPR = adjusted prevalence proportion ratio. SAH = self-assessed health.

Maternal depression was associated with higher aPPR of daily health complaints in the offspring for all depression categories (adjusted analyses, Table 3). Children of mothers categorised with first-time treatment for maternal depression more often reported daily somatic complaints (aPPR 1.49, 95% confidence interval [CI] = 0.80 to 2.76), daily mental complaints (aPPR 1.45, 95% CI = 1.00 to 2.09), and both mental and somatic complaints (aPPR 2.38, 95% CI = 1.06 to 5.35). The aPPR of any complaint was statistically significantly higher for children of mothers treated for continued depression (aPPR 1.59, 95% CI = 1.37 to 1.85), children of mothers after cessation of treatment for depression (aPPR 1.30, 95% CI = 1.20 to 1.41), and children of mothers with relapse (aPPR 1.56, 95% CI = 1.35 to 1.79) compared to children of mothers without depression. Children of mothers with depression also had higher aPPR of reporting low SAH for first-time treatment (aPPR 1.58, 95% CI = 0.99 to 2.54), continued treatment (aPPR 1.86, 95% CI = 1.51 to 2.28), post-treatment (aPPR 1.34, 95% CI = 1.19 to 1.50), and relapse treatment (aPPR 1.56, 95% CI = 1.26 to 1.93) compared to children of mothers without depression (Table 3).

### Sub-analysis

No statistically significant effect modification was seen in the sub-analysis stratified on sex (data not shown).

## DISCUSSION

### Summary

This study found that pre-adolescents of mothers with depression more often reported daily health complaints and low SAH compared to pre-adolescents of mothers without depression. This association was strongest for children of mothers with continued depression or relapse, followed by children of mothers after cessation of treatment for depression.

### Strengths and limitations

This study was based on a large population. The authors addressed potential sources of selection bias by applying IPW, which increased the generalisability of findings. A strength was that outcomes were based on self-report from the offspring, as parental reports on behavioural problems in their offspring can be overestimated when a parent is depressed.^28^^–^^30^ The design does not allow estimation of causal association between maternal depression and child outcomes. The register-based design limits the authors’ definition of depression. Mothers treated with alternative medicine or psychotherapy and women with no treatment might have been misclassified as not depressed in this study, which may have led to an underestimation of the results. Although the authors included a number of covariates in their model, residual confounding cannot be ruled out, which is a general limitation in observational studies. Moreover, the authors cannot distinguish between whether their findings reflect the child’s reaction to maternal depression or the emergence of inherited mental or somatic illness in the child.

### Comparison to existing literature

In contrast to the present study, most previous studies were based on maternal reports of offspring health complaints and psychological problems. The authors’ findings are in line with a population-based study, which found that children of parents with mental health problems had 43%–74% higher odds of asthma, abdominal pain, and headache compared to children of parents without a history of mental health problems.^25^ However, as analyses were conducted on parents jointly, no detailed results were presented for mothers. Another study found maternal depression to be associated with functional abdominal pain in a youth population with a mean age of 11.8 years, a population that is similar to that in the current study.^24^

Findings from previous studies using parental reports show strong association between maternal depression and behavioural and psychological difficulties in childhood.^42^^–^^46^ Mental health in parents has also been found to be strongly associated with level of SAH in the offspring.^22^ A twin study found that only concurrent maternal depression had significant association with behavioural problems in the offspring.^43^ In contrast, the present study found significant associations between daily health complaints and low SAH in the offspring and maternal depression beyond the initial onset of treatment — offspring of mothers with depression more often reported daily health complaints, even when the mother had received treatment for years or had ended treatment. The authors would have expected first-time treatment and relapse to hold the strongest association due to expected load of depression symptoms in this group. Surprisingly, the association was strongest for children of mothers in continued treatment for depression, suggesting that having a mother treated for depression for a long time has high impact on the child. The present study cannot disentangle the reasons behind this finding, but it suggests greater long-term consequences of maternal depression on the offspring’s subjective health perception. The children may also be genetically predisposed, and the health complaints could be preliminary symptoms of psychiatric or somatic disease in the children. Previous research found that children of mothers with depression had higher risk of not attending routine childcare,^47^ but also higher rates of non-routine contacts to primary health care in early childhood.^48^ One study performed on the same population as in this study, found higher primary health care use in children with frequent health complaints and low SAH.^31^

### Implications for practice

Frequent somatic complaints in childhood warrant concern because research points to higher risk of physical and mental disabilities later in life.^24^^,^^25^^,^^37^^,^^46^^,^^49^ Impaired functional somatic symptoms are correlated with increased primary healthcare costs.^50^ GPs could use this knowledge in encounters with families struggling with depression and children with frequent health complaints. Children with persisting unexplained somatic and mental health complaints are at risk of receiving suboptimal care and support.^51^ When a person has depression or a child has health complaints the first healthcare personnel to see them in Denmark is a GP. GPs manage the treatment of most people with depression. Furthermore, GPs are the only healthcare personnel to see a child continuously from birth to adulthood. Daily health complaints in children might be interpreted as an indication of dysfunction in other areas of life, such as in family dynamics. Likewise, during management of a mother with depression, GPs might use this awareness to discover children with daily health complaints. These findings suggest that awareness of not only concurrent, but any, history of depression may be useful in managing children with daily health complaints. A recent Danish study showed that the vast majority of GPs found it relevant to address children of parents with depression — 73% of female GPs and 63% of male GPs dealt with topics such as wellbeing of the children during consultation with mothers with depression; around 40% found their knowledge about potential consequences of parental depression for the children were limited and wanted to learn more about this area.^52^ These new findings point to maternal depression as an important factor for health perception and wellbeing in offspring.

## References

[1] Berntsson LT, Kohler L (2001). Long-term illness and psychosomatic complaints in children aged 2–17 years in the five Nordic countries. Comparison between 1984 and 1996. Eur J Public Health.

[2] Dey M, Jorm AF, Mackinnon AJ (2015). Cross-sectional time trends in psychological and somatic health complaints among adolescents: a structural equation modelling analysis of ‘health behaviour in school-aged children’ data from Switzerland. Soc Psychiatry Psychiatr Epidemiol.

[3] World Health Organization Regional Office for Europe (2016). Growing up unequal: gender and socioeconomic differences in young people’s health and well-being.

[4] Rasmussen M, Pedersen TP, Due P (2015). [The investigation of school children].

[5] Steinbrecher N, Koerber S, Frieser D, Hiller W (2011). The prevalence of medically unexplained symptoms in primary care. Psychosomatics.

[6] Liu J, Chen X, Lewis G (2011). Childhood internalising behaviour: analysis and implications. J Psychiatr Ment Health Nurs.

[7] Meilstrup C, Ersbøll AK, Nielsen L (2015). Emotional symptoms among adolescents: epidemiological analysis of individual-, classroom- and school-level factors. Eur J Public Health.

[8] Hoftun GB, Romundstad PR, Zwart JA, Rygge M (2011). Chronic idiopathic pain in adolescence — high prevalence and disability: the young HUNT study 2008. Pain.

[9] Hotopf M, Carr S, Mayou R (1998). Why do children have chronic abdominal pain, and what happens to them when they grow up? Population based cohort study. BMJ.

[10] Fearon P, Hotopf M (2001). Relation between headache in childhood and physical and psychiatric symptoms in adulthood: national birth cohort study. BMJ.

[11] Campo JV, Di Lorenzo C, Chiappetta L (2001). Adult outcomes of pediatric recurrent abdominal pain: do they just grow out of it?. Pediatrics.

[12] Homlong L, Rosvold EO, Bruusgaard D (2015). A prospective population-based study of health complaints in adolescence and use of social welfare benefits in young adulthood. Scand J Public Health.

[13] Creed FH, Davies I, Jackson J (2012). The epidemiology of multiple somatic symptoms. J Psychosom Res.

[14] Breidablik H-J, Meland E, Lydersen S (2008). Self-rated health during adolescence: stability and predictors of change (Young-HUNT study, Norway). Eur J Public Health.

[15] Simon JG, De Boer JB, Joung IMA (2005). How is your health in general? A qualitative study on self-assessed health. Eur J Public Health.

[16] Jylhä M (2009). What is self-rated health and why does it predict mortality? Towards a unified conceptual model. Soc Sci Med.

[17] Au N, Johnston DW (2014). Self-assessed health: what does it mean and what does it hide?. Soc Sci Med.

[18] Burström D, Fredlund P (2001). Self-rated health: is it as good a predictor of subsequent mortality among adults in lower as well as in higher social classes?. J Epidemiol Community Health.

[19] Lokke Vie T, Ove Hufthammer K, Lingaas Holmen T (2017). Is self-rated health in adolescence a predictor of prescribed medication in adulthood? Findings from the Nord Trøndelag Health Study and the Norwegian Prescription Database. SSM Popul Health.

[20] Hetlevik Ø, Meland E, Hufthammer KO (2020). Self-rated health in adolescence as a predictor of ‘multi-illness’ in early adulthood: a prospective registry-based Norwegian HUNT study. SSM Popul Health.

[21] Vie TL, Hufthammer KO, Meland E, Breidablik HJ (2019). Self-rated health (SRH) in young people and causes of death and mortality in young adulthood. A prospective registry-based Norwegian HUNT-study. SSM Popul Health.

[22] Petanidou D, Mihas C, Dimitrakaki C (2014). Selected family characteristics are associated with adolescents’ subjective health complaints. Acta Paediatr.

[23] Kingston D, Tough S (2014). Prenatal and postnatal maternal mental health and school-age child development: a systematic review. Matern Child Health J.

[24] Campo JV, Bridge J, Lucas A (2007). Physical and emotional health of mothers of youth With functional abdominal pain. Arch Pediatr Adolesc Med.

[25] Feldman JM, Ortega AN, Koinis-Mitchell D (2010). Child and family psychiatric and psychological factors associated with child physical health problems: results from the Boricua youth study. J Nerv Ment Dis.

[26] Homlong L, Rosvold EO, Sagatun Å (2015). Living with mentally ill parents during adolescence: a risk factor for future welfare dependence? A longitudinal, population-based study. BMC Public Health.

[27] Ertel KA, Rich-Edwards JW, Koenen KC (2011). Maternal depression in the United States: nationally representative rates and risks. J Womens Health (Larchmt).

[28] Ringoot AP, Tiemeier H, Jaddoe VWV (2015). Parental depression and child well-being: young children’s self-reports helped addressing biases in parent reports. J Clin Epidemiol.

[29] van der Toorn SLM, Huizink AC, Utens EMWJ (2010). Maternal depressive symptoms, and not anxiety symptoms, are associated with positive mother–child reporting discrepancies of internalising problems in children: a report on the TRAILS study. Eur Child Adolesc Psychiatry.

[30] Madsen KB, Rask CU, Olsen J (2020). Depression-related distortions in maternal reports of child behaviour problems. Eur Child Adolesc Psychiatry.

[31] Rytter D, Rask CU, Vestergaard CH (2020). Non-specific health complaints and self-rated health in pre-adolescents; impact on primary health care use. Sci Rep.

[32] Olsen J, Meder K (2014). Better health for mother and child — The Danish National Birth Cohort (DNBC), its structure, history and aims. Norsk Epidemiologi.

[33] Pottegård A, Schmidt SAJ, Wallach-Kildemoes H (2017). Data Resource Profile: The Danish National Prescription Registry. Int J Epidemiol.

[34] Schmidt M, Schmidt SAJ, Sandegaard JL (2015). The Danish National Patient Registry: a review of content, data quality, and research potential. Clin Epidemiol.

[35] Gardarsdottir H, Egberts ACG, van Dijk L (2009). An algorithm to identify antidepressant users with a diagnosis of depression from prescription data. Pharmacoepidemiol Drug Saf.

[36] Haugland S, Wold B (2001). Subjective health complaints in adolescence — reliability and validity of survey methods. J Adolesc.

[37] Ravens-Sieberer U, Erhart M, Torsheim T (2008). An international scoring system for self-reported health complaints in adolescents. Eur J Public Health.

[38] Bøcker Pedersen C (2011). The Danish Civil Registration System. Scand J Public Health.

[39] Hernán MA, Hernández-Díaz S, Robbins JM (2004). A structural approach to selection bias. Epidemiology.

[40] Skov T, Deddens J, Petersen MR (1998). Prevalence proportion ratios: estimation and hypothesis testing. Int J Epidemiol.

[41] Landman-Peeters KMC, Ormel J, Van Sonderen ELP (2008). Risk of emotional disorder in offspring of depressed parents: gender differences in the effect of a second emotionally affected parent. Depress Anxiety.

[42] Verkuijl NE, Richter L, Norris SA (2014). Postnatal depressive symptoms and child psychological development at 10 years: a prospective study of longitudinal data from the South African Birth to Twenty cohort. Lancet Psychiatry.

[43] Gjerde LC, Eilertsen EM, Reichborn-Kjennerud T (2017). Maternal perinatal and concurrent depressive symptoms and child behavior problems: a sibling comparison study. J Child Psychol Psychiatry.

[44] Santos IS, Matijasevich A, Barros AD, Barros FC (2014). Antenatal and postnatal maternal mood symptoms and psychiatric disorders in pre-school children from the 2004 Pelotas Birth Cohort. J Affect Disord.

[45] Woolhouse H, Gartland D, Mensah F (2016). Maternal depression from pregnancy to 4 years postpartum and emotional/behavioural difficulties in children: results from a prospective pregnancy cohort study. Arch Womens Ment Health.

[46] Shelby GD, Shirkey KC, Sherman AL (2013). Functional abdominal pain in childhood and long-term vulnerability to anxiety disorders. Pediatrics.

[47] Lyngsøe BK, Vestergaard CH, Rytter D (2018). Attendance of routine childcare visits in primary care for children of mothers with depression: a nationwide population-based cohort study. Br J Gen Pract.

[48] Lyngsøe BK, Rytter D, Munk-Olsen T (2019). Maternal depression and primary healthcare use for children: a population-based cohort study in Denmark. Br J Gen Pract.

[49] Shanahan L, Zucker N, Copeland WE (2015). Childhood somatic complaints predict generalized anxiety and depressive disorders during young adulthood in a community sample. Psychol Med.

[50] Græsholt-Knudsen T, Skovgaard AM, Jensen JS, Rask CU (2017). Impact of functional somatic symptoms on 5–7-year-olds’ healthcare use and costs. Arch Dis Child.

[51] Hinton D, Kirk S (2016). Families’ and healthcare professionals’ perceptions of healthcare services for children and young people with medically unexplained symptoms: a narrative review of the literature. Health Soc Care Community.

[52] Hansen K, Kristensen O, Søgaard H, Christensen K (2018). Danish general practitioners’ professional attention to children of parents with depression. Dan Med J.

